# Expression and localization of aquaporin water channels in adult pig urinary bladder

**DOI:** 10.1111/jcmm.14256

**Published:** 2019-03-25

**Authors:** Marian Manso, Marcus J. Drake, Chris H. Fry, Myra Conway, John T. Hancock, Bahareh Vahabi

**Affiliations:** ^1^ Department of Applied Sciences, Faculty of Health and Applied Sciences University of the West of England Bristol UK; ^2^ School of Physiology, Pharmacology and Neuroscience, Faculty of Biomedical Sciences University of Bristol, University Walk Bristol UK

## 1 INTRODUCTION

The bladder is lined by urothelium, a transitional epithelium, which is generally considered to be a poorly permeable urine‐blood barrier with a crucial function to separate tissues of the urinary tract from the noxious composition of urine.^1^ Although it is believed that kidneys are responsible for the final concentration and volume of urine, significant in vivo reabsorption and secretion of Na^+^, K^+^, urea and creatinine has been measured in rabbit and rat bladders,^1^, ^2^ as well as difference in urine composition between the renal pelvis and voided urine in human subjects, indicating net water uptake.^3^

Recent studies have shown that the urothelium expresses transmembrane water channels, aquaporins (AQPs). Currently 13 AQP (0‐12) subtypes have been identified in mammalian tissues, and from these subtypes, AQP3, AQP4, AQP7 and AQP9 have been found in the human urothelium^4^ and AQP1, AQP2 and AQP3 in rat urothelium,^5^ indicating that AQPs could regulate urothelial cell volume and osmolarity, determining the final composition of urine.

Although AQPs have been identified in normal human urothelial cells, their exact functional role requires further investigation. The adult pig bladder offers a viable animal model as it has comparable structural and physiological properties to the human bladder.^6^, ^7^ We report the initial stage to characterize the expression and localization of AQPs in adult pig urinary bladder.

## 2 MATERIALS AND METHODS

### 2.1 Tissue preparations

Bladders were dissected from ~6‐months old female pigs (*Sus scrofa domestica*), obtained at a local abattoir (Langford Abattoir, University of Bristol, Bristol, UK). Tissues were placed in ice‐cold physiological Kreb's solution gassed with 95%O_2_/5%CO_2_ (pH 7.4). From each bladder dome, urothelial cell suspension and mucosa samples (urothelium and lamina propria) were obtained. Urothelial cell suspension (30‐50 mg tissue) was obtained by gentle scraping of the luminal surface of the bladder with a scalpel blade and suspending the cells in the RNA lysis buffer. Strips of urothelium plus lamina propria (mucosa) were dissected from the anterior wall of the dome of the bladder following the natural plane of division. Mucosa samples were washed four times in PBS prior to RNA extraction.

### 2.2 RNA isolation and AQP transcription

Tissue samples were homogenized in RNA lysis buffer. Total RNA was extracted from mucosa and urothelium of 25 pig bladders using Promega SV total RNA isolation kit according to the manufacturer's instructions. cDNA samples were first amplified for Glyceraldehyde 3‐phosphate dehydrogenase (GAPDH) as a housekeeping gene (Table 1). Sterile water replaced cDNA template as a negative control. Samples were then amplified using primers specific to AQPs 1‐11 (Invitrogen, UK, Table 1). PCR was carried out as: 5‐min denaturation at 94°C; 35 cycles of 94°C for 30 s; annealing temperature for 1‐min (different annealing temperatures, Table 1); 1‐min at 70°C; 5‐min final extension at 70°C. Amplified PCR products were separated by agarose gel electrophoresis and sequenced (Eurofin MWG, Germany).

**Table 1:** Primer sequences, gene accession numbers, product size and the corresponding annealing temperatures for porcine aquaporins (AQPs) and GAPDH

Name of primer/Accession number	Forward primer (5′‐3′)	Reverse primer (5′‐3′)	PS (bp)	AT (°C)
AQP1 (NM_214454.1)	AGCTGCCAGATCAGTGTCCT	CCAGTGGTCCTGGAAGTTGT	375	60.9
AQP2 (NM_001128476.1)	GCTGCCATGTCTCCTTTCTC	TCATGGAGCAGCCAGTGTAG	318	59.0
AQP3 (NM_001110172.1)	GGGACCCTTATCCTCGTGAT	AGAAGCCATTGACCATGTCC	394	58.2
AQP4 (NM_001110423.1)	TTGCTTTGGACTCAGCATTG	TGACATCAGTCCGTTTGGAA	332	57.5
AQP5 (NM_001110424.1)	GAAGGAGGTGTGCTCTCTGG	CGTGTTGTTGTTGAGCGAGT	373	59.5
AQP6 (NM_001128467.1)	TGGATGACTGTCAGCAAAGC	CCTCAGGTATGACCCCGTAA	316	58.3
AQP7 (NM_001113438.1)	AGAGTTCTTGGCCGAGTTCA	ACCGGTCACTGTCAGCTTTC	346	59.6
AQP8 (NM_001112683.1)	GCCTGTCGGTCATTGAGAAT	GGATGATCTCTGCCACCACT	333	58.6
AQP9 (XM_005659551.1)	TGCATTTGCAGACCAGGTAG	CTGGTTTGTCCTCCGATTGT	380	58.7
AQP10 (NM_001128454.1)	TTGTGCTCATGCTCTTCACC	GGATAGGTGGCAAAGATGGA	355	57.7
AQP11 (NM_001112682.1)	CGCTTTCGTCTTGGAGTTTC	GGAGCAGATGGCCTCTATCA	388	58.4
GAPDH (XM_005658673.2)	CACGTTGGGGGTGGGGACAC	ACCCAGAAGACTGTGGATGG	171	60.0

AT, annealing temperature; bp, base pairs; PS, product size.

### 2.3 Immunohistochemistry

Fixed bladder samples from pigs (n = 12) were processed and embedded in paraffin. Tissue sections (3 μm), placed on (3‐Aminopropyl) triethoxysilane coated slides, were blocked for endogenous peroxidase activity by incubation in 0.3% H_2_O_2,_ prepared in 100% methanol (30‐min at ~20°C). Slides were then placed in sodium citrate antigen retrieval buffer followed by incubation with the specific primary antibody (AQP1, 3, 9 and 11 polyclonal antibodies, 1:1000‐1:1500 dilutions, Almone, Israel) overnight at 4°C. Negative controls replaced the primary antibody with the relevant peptide.

## 3 RESULTS

### 3.1 Identification of AQPs 1, 3, 9 and 11 transcripts in adult pig bladder

**Figure 1:**
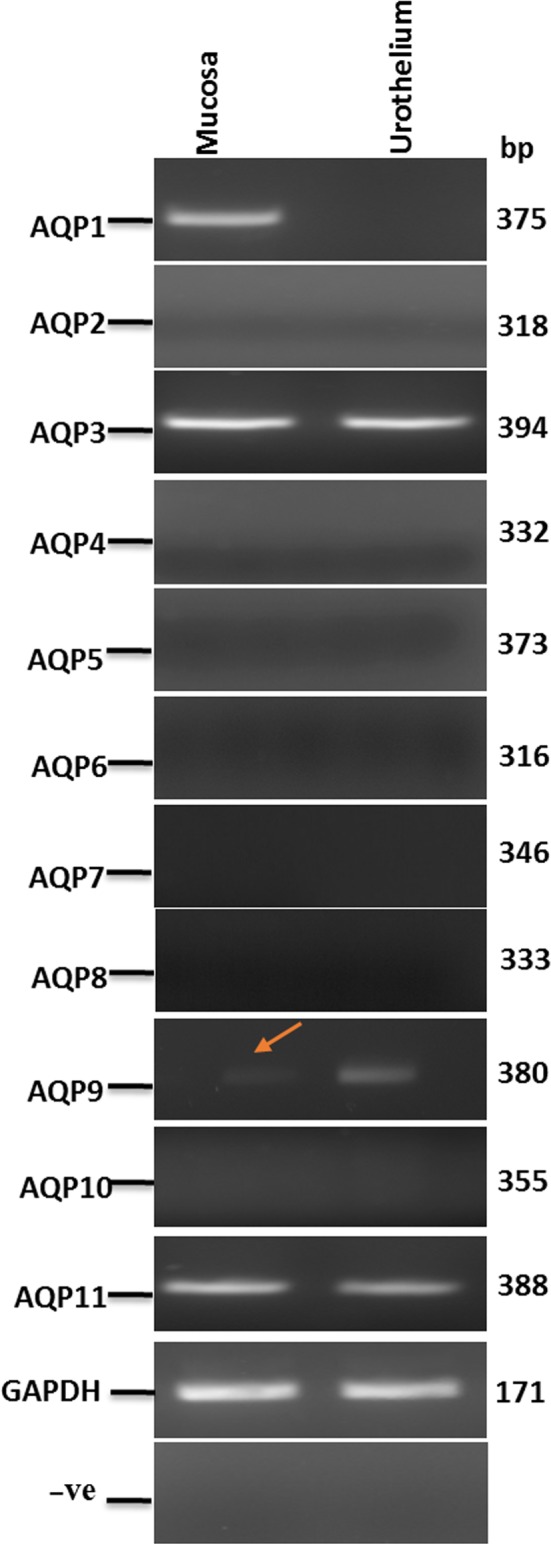
Aquaporins (AQP) transcript expression in pig bladder. Expression of AQP1‐11 transcripts in a representative pig bladder mucosa and the corresponding urothelium sample; the GAPDH band is shown below. The negative controls are where nuclease‐free water substituted reverse transcriptase in the amplification process

RT‐PCR demonstrated the expression of AQP1 in the mucosa but not the urothelium (Figure 1) and AQP3, AQP9 and AQP11 in both the mucosa and the urothelium of pig bladder (Figure 1). The expression of AQP2, AQP4‐8 and AQP10 could not be detected. Direct sequencing of the AQP PCR amplicons from bladder tissue yielded partial sequences (~276‐354 bp) with homology range of 97%–100% with *Sus scrofa* AQPs 1, 3, 9 and 11 (for accession numbers see Table 1).

### 3.2 Immunoperoxidase labelling of AQP1, 3, 9 and 11 in adult pig bladder

**Figure 2:**
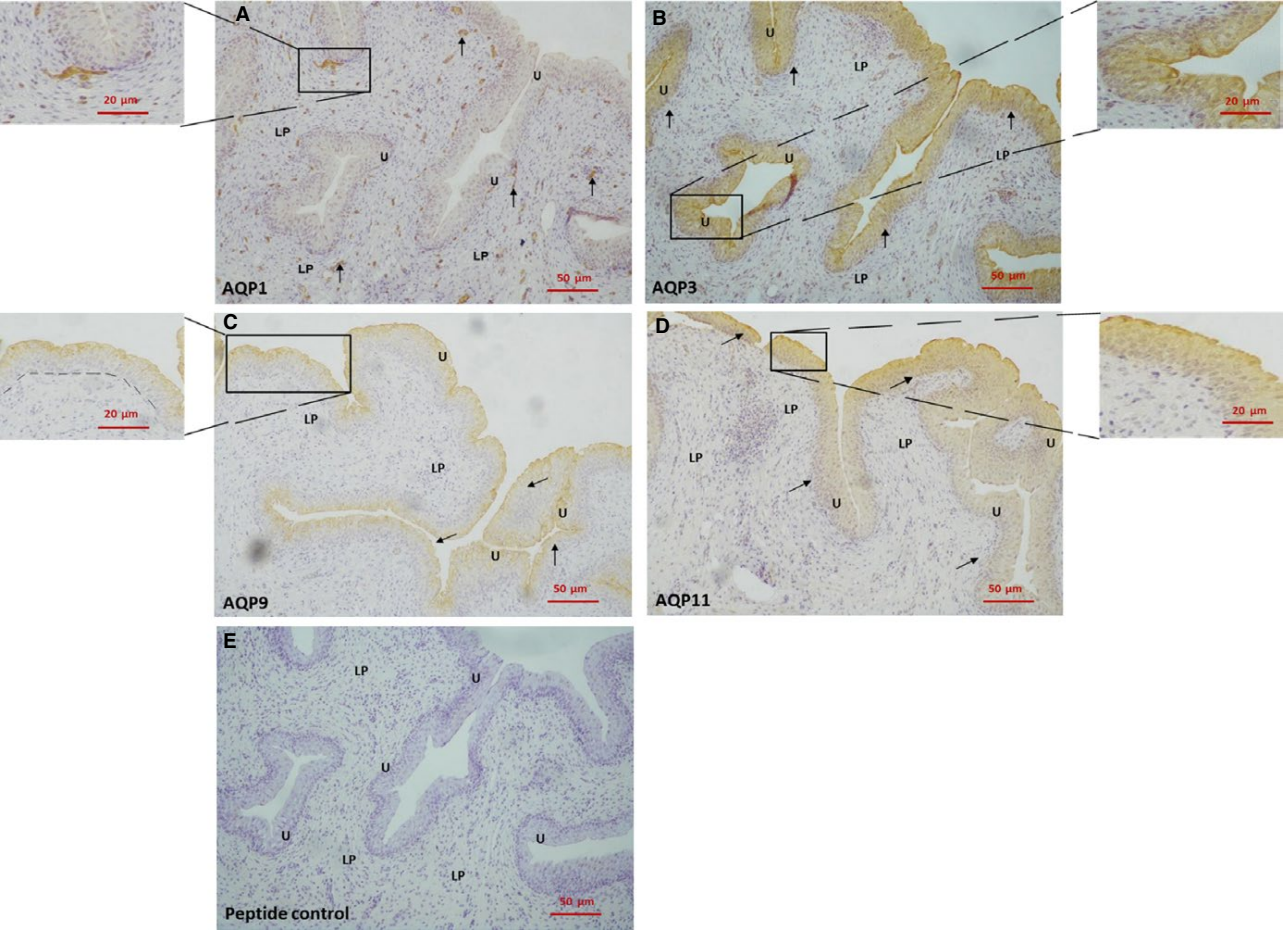
Immunoperoxidase labelling of AQPs in pig bladder. A, bladder mucosa with AQP1 immunoreactivity in the lamina propria. The inset shows a small blood vessel immediately below the urothelium at a larger magnification. B, bladder mucosa with AQP3 immunoreactivity in the urothelium. The inset shows the urothelium at a larger magnification. C, bladder mucosa with AQP9 immunoreactivity in the urothelium. The inset shows the urothelium at a larger magnification. The dotted line demonstrates the boundary between the urothelium and the lamina propria. D, bladder mucosa with AQP11 immunoreactivity in the urothelium. The inset shows the urothelium at a larger magnification. E, A representative bladder mucosa with peptide control. U, urothelium; LP, lamina propria

AQP1 immunoreactivity was present in the lamina propria, localized to endothelial cells in capillaries and arterioles (Figure 2A). AQP3 and 11 labelling was detected throughout the urothelium (Figure 2B,D). AQP9 labelling was identified in the upper region of the urothelium which would include umbrella and intermediate cells (Figure 2C). AQP1, 3, 9 and 11 peptide controls showed no labelling in the bladder (representative Figure 2E).

## 4 DISCUSSION

The aim of this study was to determine the expression profile of AQPs in the adult pig bladder as it is a convenient model to describe human bladder function. RNA expression and protein translation of AQP1, AQP3, AQP9 and AQP11 were found.

AQP1 was confined to lamina propria blood vessels, as observed in rat bladder and ureter.^5^ The functional role of AQP1 in proximal tubular and descending limb of the loop of Henlé epithelium is to mediate water fluxes. AQP1 is also expressed in mice airways and lungs where it facilitates osmotic water transport across alveolar microvascular endothelium.^8^ However, AQP1 knockout had no impact on alveolar fluid absorption, impaired humidification or lung CO_2_ transport.^8^ Similarly, deletion of AQP1 in the microvascular endothelial cells of salivary gland in mice had no effect on the secretion of saliva.^8^ It appears that AQP1 has limited physiological function in fluid movement in the endothelium, but its functional role in the microvasculature of the bladder mucosa is unknown.

AQP3 was expressed throughout the urothelium of the pig bladder, consistent with data from human and rat bladder urothelium.^4^, ^5^ Rubenwolf et al^9^ also reported that AQP3 was located especially at the intercellular borders of basal and intermediate cells, but in the pig bladder, this study demonstrated that distribution was homogeneous throughout the urothelium. AQP3 transports several neutrally charged solutes, including water, glycerol and urea. Exposure of human urothelial cells to hyperosmolar NaCl (500 mosm/kg) solutions increased AQP3 expression, followed by protein migration to the surface membrane.^9^ This suggests that AQP3 expression and migration is part of a mechanism to regulate urothelial cell osmolarity and volume during increased osmotic stress as caused by exposure to urine of variable osmolality. AQP3 is a member of the aquaglyceroporins and thus also transports urea and other solutes.^10^ Based on the similarities in the function of AQP3 and urea transporter B, which has also been found in the urothelium^11^ it may be postulated that AQP3 also facilitates the transfer of urea across the bladder urothelium.

AQP9 was detected more on the apical surface of the pig urothelium, that is umbrella and intermediate cells. Basal cells differentiate into intermediate and umbrella cells^12^ and other studies have shown that AQP9 expression is evident in differentiating keratinocytes^13^ and human urothelium cultures.^4^ AQP9 belongs to the aquaglyceroporins and a 3D‐structural analysis of APQ9 showed a larger pore size compared to other AQPs and can explain its ability to transport larger molecules such as lactate, purines and pyrimidines.^14^ Thus, AQP9 could modify the final composition of urine by facilitating transport of various solutes across the bladder urothelium.

AQP11 was identified throughout the urothelium. AQP11 is mainly present in the endoplasmic reticulum and only a fraction migrates to the cell membrane to facilitate glycerol and water transport.^15^ AQP11 expression has also been shown in human adipocytes where it functions as a water and glycerol channel.^15^ Thus, AQP11 may act as a water and glycerol transporter in bladder urothelium under osmotic stress.

In conclusion, AQP1, 3, 9 and 11 are present in adult pig bladder, suggesting that AQPs may regulate urothelium cell volume and determine the final urine composition. Changes to the extracellular osmolality may also generate membrane stress, and release various transmitters from the bladder urothelium, affecting bladder contractility and sensory nerve transduction. However, the exact role of AQPs in mediating the sensory and contractile functions of the bladder wall is unknown.

## CONFLICT OF INTEREST

The authors confirm that there are no conflicts of interest.
